# A need to reconsider guidelines on management of primary spontaneous pneumothorax?

**DOI:** 10.1186/s12245-017-0135-x

**Published:** 2017-02-21

**Authors:** Jiyoon Yoon, Parthipan Sivakumar, Kevin O’Kane, Liju Ahmed

**Affiliations:** 1 Department of Respiratory Medicine, St Thomas’ Hospital, Westminster Bridge Road, London, SE1 7EH UK; 2 Department of Emergency Medicine, St Thomas’ Hospital, Westminster Bridge Road, London, SE1 7EH UK

**Keywords:** Pneumothorax, Guidelines, Emergency medicine, Respiratory, Pleural disease

## Abstract

**Background:**

The key guidelines in the management of primary spontaneous pneumothorax (PSP) include the 2010 British Thoracic Society (BTS) Pleural Disease guideline and 2001 American College of Chest Physicians (ACCP) Consensus Statement. Current recommendations are dependent on radiographic measures which differ between these two guidelines. The aim of this study is to compare size classification of PSP cases, according to BTS and ACCP guidelines, and to evaluate guideline compliance.

**Findings:**

We conducted a retrospective evaluation of all PSP episodes presenting to St Thomas’ Hospital, London, between February 2013 and December 2014. Data was recorded from review of chest X-rays and patient records. Eighty-seven episodes of PSP in 72 patients were identified (median age 25 years, IQR 22–32.25). Classification of “large” and “small” showed the greatest disparity in those managed conservatively (12/27, 44%) or with aspiration only (11/23, 48%). In this UK study, BTS guidelines were followed in 70% of episodes with adherence to ACCP guidelines in 32% of episodes.

**Conclusions:**

There is a poor agreement in size classification between BTS and ACCP guidelines, resulting in conflicting recommendations for management of PSP. Robust clinical trial evidence is required to achieve international consensus on the management of PSP.

## Introduction

Primary spontaneous pneumothorax (PSP) is a condition that affects young, otherwise healthy people. A recent large epidemiological study reports an annual incidence of 22.7 per 100,000 and a sex ratio of 1:3.3 (women: men) [1]. Guidelines for the management of PSP include those produced by the British Thoracic Society (BTS) [2] and the American College of Chest Physicians (ACCP) [3]. A key distinction made in treatment of clinically asymptomatic patients is the size of the PSP, with ‘large’ defined as greater than 2 cm rim at the hilum (BTS) or greater than 3 cm apex-to-cupola distance (ACCP). We evaluated the classification and management of PSP according to the two guidelines in a local cohort.

## Methods

Data was collected retrospectively from Emergency Department (ED) electronic records of St Thomas’ Hospital, London. All patients diagnosed with pneumothorax, between February 2013 and December 2014, were included, with exception of individuals over 50 years with significant smoking history (>20 pack years and current smokers, where records were unclear), or underlying lung disease (not including previous pneumothorax). Immediate recurrence was defined as a re-attendance within 30 days of the first episode. Guideline adherence is defined in Table 1. Electronic patient records, including admission notes, chest radiographs, dates of admission and follow-up clinic notes were used to gather data.

**Table 1:** Summary of the BTS and ACCP guidance on the management of primary spontaneous pneumothorax

	Size of pneumothorax	BTS	ACCP
Stable^a^ and asymptomatic	Small	No intervention	No intervention
	Large	1. Needle aspiration *If fails* 2. Intercostal chest drain	Intercostal chest drain
Unstable or breathless	NA	1. Needle aspiration *If fails* 2. Intercostal chest drain	Intercostal chest drain

^a^Stable fulfilling all criteria - Respiratory rate <24 breaths/min; heart rate 60–120 bpm; O2 saturation >90% on room air; blood pressure >90/60; able to complete full sentences between breaths

## Findings

Eighty-seven episodes of PSP presenting to ED in 72 patients were identified (Table 2). Median age was 25 years (IQR 22–32.25), and 62 patients were male. BTS and ACCP guidelines conflicted on size classification in 30 episodes (34%). In this UK-based study, management was predominantly in line with the BTS guidelines over ACCP, with 70% overall compliance rate.

**Table 2:** Disparity in size classification in the differing treatment groups and adherence to BTS and ACCP guidance

Treatment	Total	Classified as ‘large’ (%)		Disparity in classification (%)	BTS guidelines followed (%)	ACCP guidelines followed (%)
		BTS	ACCP			
Whole group	87	40 (46)	67 (77)	30 (34)	61 (70)	28 (32)
Conservative	27	0 (0)	11 (41)	12 (44)	27 (100)	16 (59)
Needle aspiration only	23	10 (43)	23 (100)	11 (48)	14 (61)	0^a^
Intercostal chest drain (all)	37	30 (81)	33 (89)	7 (19)	20 (54)	12 (32)
ICD with inpatient stay	15	13 (87)	14 (93)	3 (20)	7 (47)	8 (53)
ICD with outpatient management	22	17 (77)	19 (86)	4 (18)	13 (59)	4 (18)

^a^ACCP guidelines do not recommend aspiration

Twenty-seven episodes were conservatively managed, all of which were classified as small according to BTS. In 12 episodes (44%), ACCP classification conflicted with BTS. Despite this, all episodes were managed according to BTS guidelines.

Management of PSP by needle aspiration (NA) only met BTS guidelines in 14/23 episodes (61%). Disparity in classification was highest among the treatment groups, at 11/23 (48%). Those episodes that underwent needle aspiration contrary to BTS guidance (classified as ‘small’) had ‘large’ pneumothoraces according to ACCP guidelines.

Fifteen episodes were managed with standard intercostal chest drain (ICD) insertion; 7/15 episodes (47%) were compliant with BTS guidelines. Eight cases were non-compliant due to insertion of drain without prior NA. Of these, 5 were symptomatic or clinically unstable.

In 22 cases, an ambulatory device (Atrium pneumostat) was attached to a standard ICD. Of these, 18 underwent standard drain insertion, which was subsequently attached to an ambulatory device during admission to allow early discharge. Four cases were ambulated in the first instance. Although ambulatory management does not feature in the guidelines, the indication for initial ICD insertion followed BTS guidelines in 13 of 22 episodes (59%). Of the remaining 9 episodes, 2 had ‘small’ PSP according to BTS but were classified as ‘large’ according to ACCP and underwent ICD insertion. Two episodes had ‘small’ PSP according to both guidelines but had history of recurrent PSP and previous pleurodesis and had ICDs inserted. Five had ‘large’ PSP and did not undergo NA prior to ICD insertion; four of these episodes were symptomatic.

## Discussion

Significant deviation from BTS guidelines can be seen throughout the groups studied. We observe two major patterns of non-adherence.

Firstly, NA is frequently omitted in favour of ICD in ‘large’ PSP. Reasons for deviation are unclear but may be due to physicians choosing to pursue a ‘single definitive procedure’. Overall, the management of 26 PSP episodes deviated from BTS guidelines. Among these, 9/26 (35%) were symptomatic or clinically unstable, all of which had ICD insertion with omission of NA.

Prospective data on the efficacy of NA in comparison to ICD is conflicting. Noppen et al. found no difference between NA and ICD on success of the procedure (93 and 85%, *p* = 0.4) [4]. However Andrivet et al. found significantly lower success in NA than with ICD (67 and 93%, *p* = 0.01) [5], although the authors note more stringent criteria for NA compared to ICD (declared as failure after a maximum aspiration period of 60 min vs 10-day period, respectively).

This uncertainty is reflected in the ACCP consensus statement [3], which states NA is inappropriate in most cases, with the exception of stable patients with a ‘small’ pneumothorax that expands during observation.

Although data on efficacy of NA differ, studies suggest that given minimal complications, possible decrease in hospitalisation (52 vs 100%, *p* < 0.0001) [4], decreased pain [6], and no difference in recurrence rate [4–6], NA is a simple and safe first-line treatment for PSP.

Secondly, apical pneumothoraces are poorly represented in the size classification in BTS guidelines. All cases non-compliant with guidelines in the NA group had ‘small’ PSP according to BTS but ‘large’ according to ACCP, which favours apical pneumothorax identification.

Collins et al. quantified the difference in size of PSP classified as ‘large’ under different international guidelines using the volumetrically-derived Collins’ method [7]. The median size of ‘large’ PSP was estimated to be 95 and 31% of the volume of the hemithorax, for BTS and ACCP, respectively [8].

Our data shows marked disagreement in size classification within the group that is conservatively managed which may reflect the impact of clinical judgement. Given that size classification shows such disparity, it is likely that many patients with ‘large’ PSP, by either standard, are routinely being managed conservatively without adverse consequences. This emphasises the importance of clinician experience and sound decision making in the safe management of PSP. Despite this, there is a paucity of high-quality data regarding the efficacy and safety of conservative vs interventional treatment of PSP, with a recent Cochrane review finding no admissible studies for meta-analysis [9]. Perhaps a study comparing symptom directed management with current guidance would be suitably placed to answer this question.

In our study, we also included patients treated on an ambulatory basis, a pathway currently being prospectively evaluated for safety and efficacy [10]. Whilst guidelines cannot aim to encompass every clinical scenario, especially in the light of new developments, there is currently lack of standardisation. This not only affects clinical practice but also limits availability of comparable data.

Fundamental to dealing with uncertainty is acknowledging the limitation of conventional wisdom. Before guidelines can be revised, there is a case to be made for seeking robust clinical trial evidence, a scientific endeavour currently undertaken by the Randomised Ambulatory Management of Primary Pneumothorax study (RAMPP) [10]. Then only may we reach an elegant international consensus with which to direct management.

## Abbreviations

ACCP: American College of Chest Physicians
BTS: British Thoracic Society
PSP: Primary Spontaneous Pneumothorax
